# Supplementary material for: Decision Regret and Vaccine Hesitancy among Nursing Students and Registered Nurses in Italy: Insights from Structural Equation Modeling
Source: Vaccines (Basel). 2024 Sep 14;12(9):1054. doi: 10.3390/vaccines12091054 (PMC11435976; doi:10.3390/vaccines12091054)
Supplement: Supplementary file 1 [file vaccines-12-01054-s001.zip › Supplementary File S1.pdf]

Supplementary File S1. Translation into Italian and validation of the Decision Regret Scale (DRS)

The Decision Regret Scale (DRS) is a widely used tool for measuring regret associated with healthcare decisions. Numerous studies have consistently validated its unidimensionality, confirming that the scale measures a single underlying construct of decision regret. In this study, we confirmed the unidimensionality of the DRS following its translation into Italian. To ensure the validity of the translated items, a content validity assessment was conducted using the Content Validity Ratio (CVR). This method involved expert evaluations to ascertain the relevance and clarity of each item in the Italian context.

<u>TRANSLATION PROCESS</u>

The translation of the DRS into Italian followed a structured process to ensure the accuracy and validity of the translated instrument. The process adhered to the collaborative and iterative translation approach proposed by Douglas and Craig (2007)[1], which emphasizes cultural and conceptual equivalence over a simple literal translation. The translation team consisted of two independent bilingual translators who were native speakers of Italian and fluent in English. Each translator produced a separate version of the DRS, ensuring diverse linguistic perspectives. A synthesis meeting was conducted with the two translators and a bilingual reviewer to compare the independent translations. The team aimed to resolve discrepancies and agree on a unified version of the DRS that best captured the intended meaning of the original items. The review and adjudication process involved a committee of five experts in translation, healthcare decision-making, and psychometrics. This committee reviewed each item, focusing on cultural nuances and conceptual equivalence.

In detail, the translation process involved several key stages, each designed to ensure that the Italian version of the DRS would be both accurate and meaningful within the Italian cultural context.

1. Initial Translation

The initial translation was conducted by two independent bilingual translators who are native speakers of Italian. Each translator produced a separate version of the DRS, ensuring a diverse linguistic perspective.

2. Synthesis of Translations

A synthesis meeting was held where the two translators, along with a bilingual reviewer, compared the independent translations. The goal was to identify discrepancies and agree on a single, unified version of the DRS that best captured the intended meaning of the original items.

3. Review and Adjudication

A committee comprising experts in translation, healthcare decision-making, and psychometrics reviewed the synthesized translation. This committee discussed each item in detail, focusing on cultural nuances and conceptual equivalence. Adjustments were made to ensure that the translated items were clear and relevant to Italian respondents.

Results of Translated Items:

1. **Original Item:** "It was the right decision."
    - **Translated Item:** "È stata la decisione giusta."

[1] Douglas, Susan P., and C. Samuel Craig. "Collaborative and iterative translation: An alternative approach to back translation." *Journal of International Marketing* 15.1 (2007): 30-43.

- **Comment:** The committee agreed that this translation accurately conveys the intended meaning and is culturally appropriate for Italian respondents.

2. **Original Item:** "I regret the choice that was made."
   - **Translated Item:** "Rimpiango la scelta che ho fatto."
   - **Comment:** The translation was deemed accurate and clear, reflecting the emotional weight of the original item.
3. **Original Item:** "I would go for the same choice if I had to do it over again."
   - **Translated Item:** "Se potessi tornare indietro rifarei la stessa scelta."
   - **Comment:** The committee found this translation to be a faithful representation of the original item's intent and meaning.
4. **Original Item:** "The choice did me a lot of harm."
   - **Translated Item:** "La scelta mi ha molto danneggiato."
   - **Comment:** This translation was considered clear and appropriate, effectively conveying the negative impact described in the original item.
5. **Original Item:** "The decision was a wise one."
   - **Translated Item:** "È stata una scelta saggia."
   - **Comment:** The translation captures the positive evaluation of the decision, aligning well with the original item's meaning.

4. Content Validity Assessment

To further validate the content, the Content Validity Ratio (CVR) was used. A panel of 10 subject matter experts evaluated the relevance and clarity of each item in the translated DRS. Items that did not meet the CVR threshold were revised or removed. The panel included six females and four males, with an age range of 30 to 65 years. Their professional backgrounds were diverse, comprising three experts in healthcare decision-making, two experts in data analysis, three clinical experts from the medical field (physicians), and two clinical nurses. The experts had between 5 and 25 years of experience in their respective domains. Their qualifications included five PhDs, three Master's degrees, and two Bachelor's degrees.

Each expert rated the relevance of each item on a scale from 1 to 3, where 1 indicates "not relevant," 2 indicates "somewhat relevant," and 3 indicates "highly relevant." The CVR for each item was then calculated using the formula: $CVR = (n_e - N/2)/(N/2)$, where n_e is the number of experts rating the item as highly relevant (rating of 3), and N is the total number of experts. For 10 raters, the critical CVR value at a 0.05 significance level is approximately 0.62. This means that a CVR value must be equal to or greater than 0.62 to be considered valid.

CVR Description

Item	Ne	CVR	Interpretation
Item 1	10	1.0	Essential
Item 2	10	1.0	Essential
Item 3	10	1.0	Essential
Item 4	10	1.0	Essential
Item 5	9	0.8	Essential

The content validity assessment, conducted using the CVR, confirmed that all items in the Italian translation of the DRS are valid. This rigorous validation process ensures that the translated scale accurately measures decision regret in Italian-speaking populations, maintaining the integrity and reliability of the original instrument.

5. Pretesting and Cognitive Debriefing

The preliminary Italian version of the DRS was pretested with a sample of 5 Italian-speaking individuals. Cognitive debriefing sessions were conducted to gather feedback on the clarity, comprehensibility, and relevance of the items. Participants were asked to explain their understanding of each item, which provided insights into potential issues.

Pretesting Results:

- Item 1: Participants clearly understood the item as intended, confirming its clarity and relevance.
- Item 2: Participants expressed a clear understanding of the item, indicating it accurately reflects decision regret.
- Item 3: Participants confirmed the item was comprehensible and relevant, reflecting their willingness to repeat the decision.
- Item 4: Participants understood the item well, confirming it conveyed the intended negative impact.
- Item 5: Participants found the item clear and relevant, accurately reflecting the wisdom of the decision.

Based on these insights, minor adjustments were made to enhance the clarity of specific items, ensuring that the final version was both linguistically accurate and culturally appropriate.

<u>CFA</u>

The CFA was performed using the dataset containing variables identified as profile (ID variable) and items i1 to i5. For the purpose of this analysis, only the variables i1, i2, i3, i4, and i5 were used, as they represent the items of the DRS.

The analysis employed the Maximum Likelihood Robust (MLR) estimator. This estimator was chosen for its robustness to non-normality, providing reliable standard errors and chi-square test statistics even when the data does not follow a normal distribution. The analysis was set to run for a maximum of 1000 iterations, allowing sufficient attempts for the optimization process to converge to a solution. The convergence criterion was set at 0.00005, indicating a high level of precision required for the parameter estimates.

In specifying the model, the DRS was defined as a latent variable measured by the five observed items (i1 to i5). This model structure was articulated as "DRS BY i1 i2 i3 i4 i5," signifying that the latent construct DRS is represented by these five items.

For the output, standardized estimates were requested to facilitate interpretation of the results. Additionally, technical output related to the estimation process was included to provide deeper insights into the model fitting. Modification indices for parameters with a chi-square value of at least 10 were also requested, helping identify potential areas of model misfit and guiding possible model improvements.

#code

```
TITLE: Validity of Decision Regret Scale (DRS) - CFA

DATA:

  FILE IS DRS.dat;

VARIABLE:

  NAMES ARE profile i1-i5;

  USEVARIABLES ARE i1 i2 i3 i4 i5;

ANALYSIS:

  ESTIMATOR = MLR;

  ITERATIONS = 1000;

  CONVERGENCE = 0.00005;

MODEL:

  DRS BY i1 i2 i3 i4 i5;

OUTPUT:

  stdyx;

  tech4;

  mod(10);
```

The model well explained sample statistics: $\chi^2(5, N=324) = 10.636$, p = 0.0591; RMSEA = 0.059, 90%CI(0.000-0.109), p = 0.322; CFI = 0.980; TLI = 0.960; SRMR = 0.034

Item	Estimate	S.E.	Est./S.E.	P-Value
DRS BY i1	0.880	0.035	25.084	<0.001
DRS BY i2	-0.666	0.051	-13.007	<0.001
DRS BY i3	0.888	0.026	34.658	<0.001
DRS BY i4	-0.703	0.046	-15.298	<0.001
DRS BY i5	0.853	0.027	31.113	<0.001

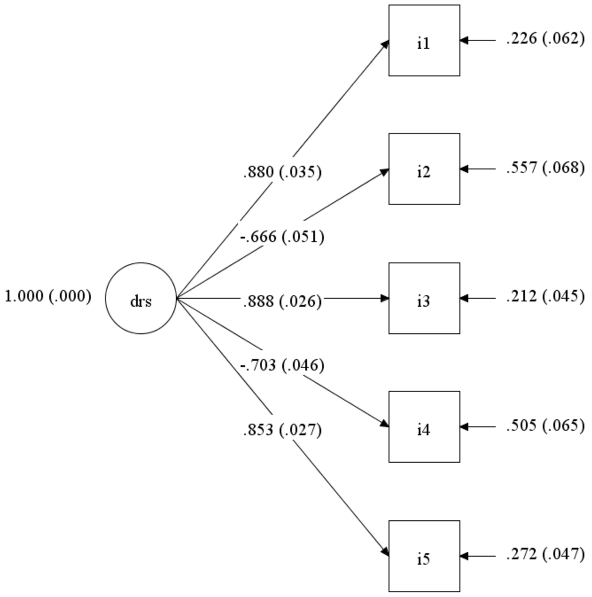

Based on the standardized estimates, items i2 and i4 have negative factor loadings, indicating that these items measure regret in the opposite direction compared to the other items. Therefore, items i2 and i4 should be reverse-coded to ensure consistency in the direction of scoring. This step is essential to accurately reflect the level of decision regret, where higher scores indicate more regret. The Omega reliability coefficient for the Italian version of the DRS, considering the reverse-coded items, is 0.899 and Cronbach's alpha is 0.896.
