# Supplementary material for: Decision Regret and Vaccine Hesitancy among Nursing Students and Registered Nurses in Italy: Insights from Structural Equation Modeling
Source: Vaccines (Basel). 2024 Sep 14;12(9):1054. doi: 10.3390/vaccines12091054 (PMC11435976; doi:10.3390/vaccines12091054)
Supplement: Supplementary file 1 [file vaccines-12-01054-s001.zip › Supplementary File S2.pdf]

Supplementary File S2. Dimensionality of the Adult Vaccine Hesitancy Scale (aVHS)

Despite the reliability of the scale being previously confirmed in the Italian version of the aVHS, its dimensionality had not been assessed.[1] Considering the preliminary dimensionality testing of the developmental article, it was necessary to perform an Exploratory Factor Analysis (EFA) exploring from a one-factor solution to a three-factor solution.[2] The decision on the number of factors to extract was based on the following criteria: a parallel analysis performed in R, previous evidence of dimensionality, interpretation of the EFA models in terms of the absence of cross-loadings, and the straightforward interpretation of the relationships between observed variables and their factors. This exploration of the dimension was performed to guide the scoring procedure of the scale, which was determined to be the mean of the items retained for each factor. Interpreting the wording of items within each factor allows the labeling of the factors.

PARALLEL ANALYSIS

library(psych)

library(foreign)

data <- read.table("path\data.dat", header = TRUE)

parallel <- fa.parallel(data, fa = "both", n.iter = 100, show.legend = FALSE, main = "Parallel Analysis") # This function performs the parallel analysis. The argument fa = "both" specifies that both principal components analysis (PCA) and factor analysis (FA) eigenvalues are computed. The n.iter = 100 argument specifies the number of iterations to generate random datasets, which is typically set to 100. The plot generated by fa.parallel helps to determine the number of factors to retain. The number of factors is determined by the point at which the eigenvalues from the actual data intersect or fall below the eigenvalues from the randomly generated data.

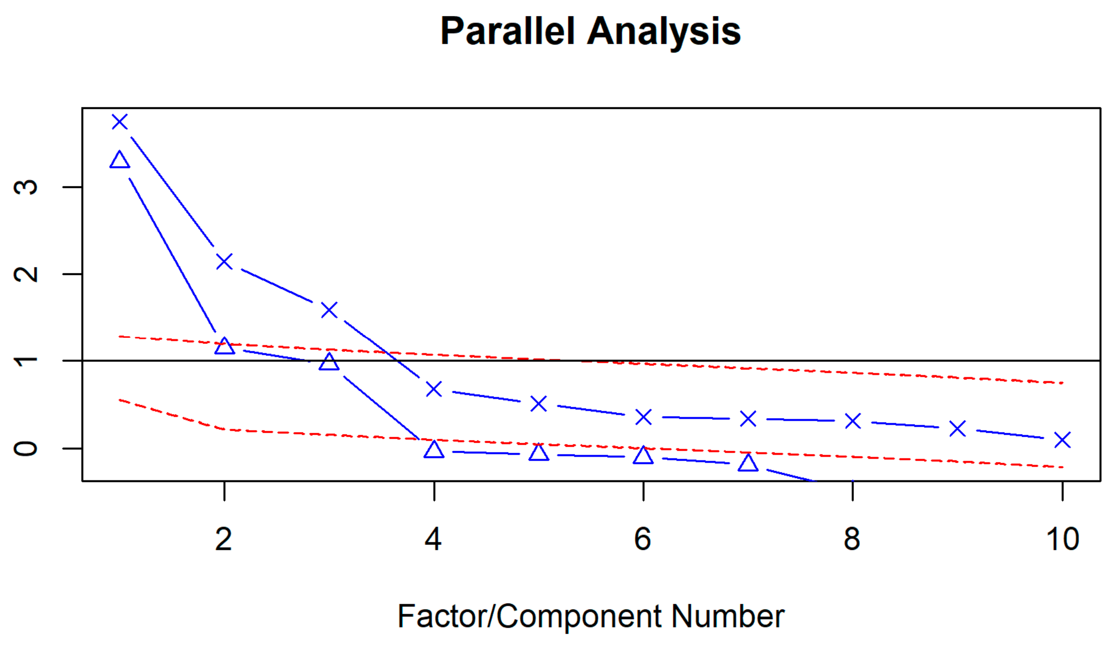

[1] Ledda, C.; Costantino, C.; Liberti, G.; Rapisarda, V. The Italian Version of the Adult Vaccine Hesitancy Scale (aVHS) for the Working-Age Population: Cross-Cultural Adaptation, Reliability, and Validity. Vaccines 2022, 10, 224. https://doi.org/10.3390/vaccines10020224

[2] Larson, H. J., Jarrett, C., Schulz, W. S., Chaudhuri, M., Zhou, Y., Dube, E., ... & Wilson, R. (2015). Measuring vaccine hesitancy: the development of a survey tool. *Vaccine*, *33*(34), 4165-4175.

The parallel analysis scree plot visually represents the eigenvalues derived from both the actual and randomly generated data. This analysis helps determine the appropriate number of factors to retain in a factor analysis. In the plot, the blue line with crosses represents the eigenvalues from the principal components analysis (PCA) of the real data, while the blue line with triangles shows the eigenvalues from the factor analysis (FA) of the real data. The red dotted lines indicate the 95th percentile of the eigenvalues from randomly generated data, serving as a benchmark for comparison. The critical point to observe is where the eigenvalues of the real data drop below those of the random data. This indicates the number of factors that explain more variance than would be expected by chance. Typically, eigenvalues greater than 1 suggest that a factor explains more variance than a single observed variable. This is known as the Kaiser Criterion, which is often used to determine factor retention.

In an in-depth interpretation, the first factor has an eigenvalue of approximately 4.5, significantly above the red dotted lines, indicating that the first factor explains a substantial amount of variance in the data. The second factor has an eigenvalue around 2.3, also above the red dotted lines, suggesting that a second factor is meaningful and explains additional variance. The third factor has an eigenvalue of about 1.5, still above the red dotted lines, signifying the presence of a third meaningful factor. However, the fourth eigenvalue, approximately 0.6, and the subsequent eigenvalues fall below the red dotted lines, implying that additional factors beyond the third do not explain a significant amount of variance compared to random data. Based on the parallel analysis scree plot, it is advisable to retain three factors. The eigenvalues of the real data for the first three factors are all above the corresponding eigenvalues from the random data, indicating that these factors explain meaningful variance in the dataset.

SCREE PLOT

The parallel analysis supports a three-factor solution in the context of a dubious scree plot. The scree plot provides a visual representation of the eigenvalues derived from the factor analysis. Initially, it shows a steep decline, suggesting the presence of multiple meaningful factors. However, the subsequent flattening of the curve raises uncertainty about the exact number of factors to retain.

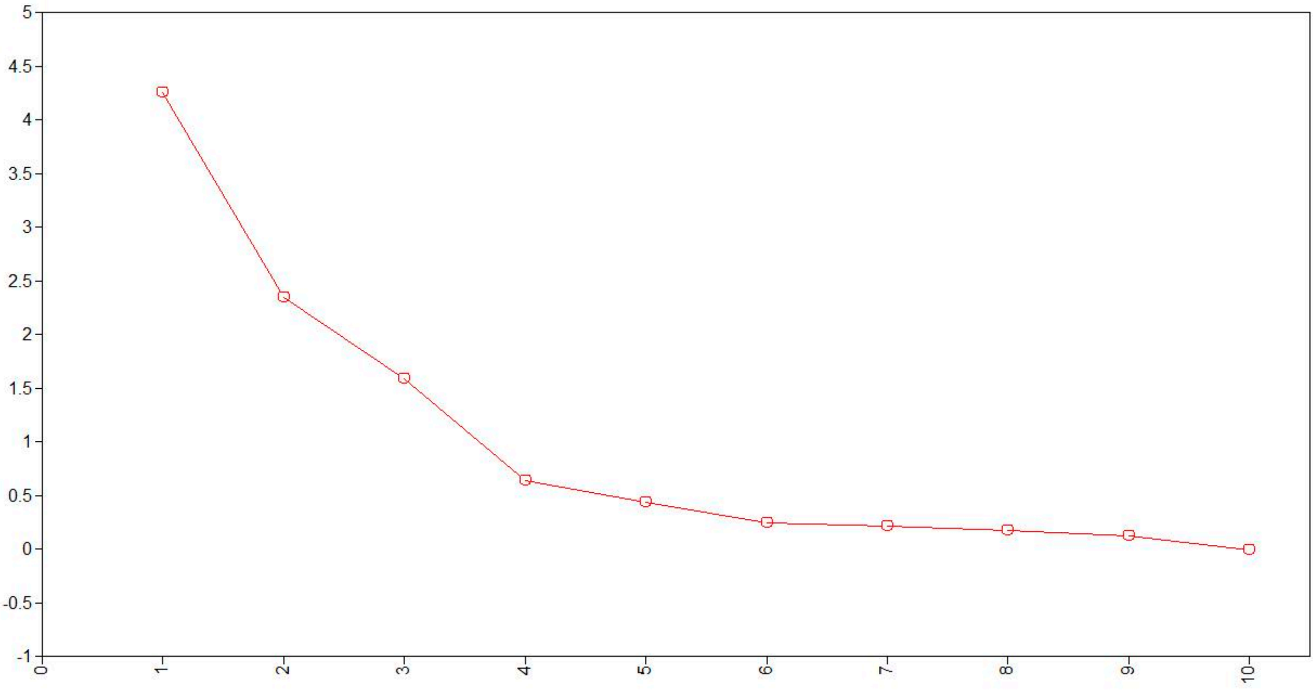

Parallel analysis offers a more robust criterion by comparing the eigenvalues of the actual data to those from randomly generated data. In this analysis, the first three eigenvalues of the real data are significantly higher than those from the random data, indicating that these three factors explain substantial variance in the dataset. Specifically, the first factor has an eigenvalue around 4.5, the second around 2.3, and the third around 1.5, all above the red dotted lines representing the 95th percentile of the eigenvalues from random data.

The fourth eigenvalue and subsequent ones fall below the threshold set by the random data, suggesting that additional factors do not contribute meaningfully beyond what could be expected by chance. This convergence of evidence from parallel analysis, despite the ambiguities in the scree plot, supports the retention of three factors for a meaningful and interpretable factor structure.

<u>EFA in MPLUS</u>

```
TITLE: Validity of VHS - CFA

DATA:
  FILE IS VHS.dat;

VARIABLE:
  NAMES ARE i1-i10;
  USEVARIABLES = i1-i10;
ANALYSIS:
  ESTIMATOR = MLR;
  TYPE = EFA 1 3;
  ROTATION = VARIMAX;

OUTPUT:
  STDYX;
  TECH4;
  MODINDICES (ALL);

PLOT: TYPE = PLOT2
```

No cross-loadings emerged from the model and its fit was optimal:

$\chi^2_{(18, N=324)} = 20.504$, $p = 0.305$; RMSEA = 0.021, 90%CI(0.000-0.056), $p = 0.906$; SRMR = 0.012

Variable	Factor 1	Factor 2	Factor 3
I1	**0.800**	0.186	0.023
I2	**0.831**	0.154	0.042
I3	**0.86**	0.195	0.000
I4	**0.787**	0.15	-0.051
I5	0.208	**0.921**	0.02
I6	0.004	0.051	**-0.682**
I7	0.023	-0.002	**-0.863**
I8	-0.032	-0.02	**-0.724**
I9	0.203	**0.932**	0.007
I10	0.114	**0.457**	-0.037

Based on the factor loadings table and the corresponding Italian version of the aVHS items, we can assign labels to the three factors as follows:

Factor 1: Trust in Vaccine Efficacy and Benefits

- I1: "I vaccini sono importanti per la mia salute" (Vaccines are important for my health)
- I2: "I vaccini sono efficaci" (Vaccines are effective)
- I3: "Essere vaccinati è importante per la salute degli altri della mia comunità" (Being vaccinated is important for the health of others in my community)
- I4: "Tutte le vaccinazioni di routine raccomandate dal Ministero sono utili" (All routine vaccinations recommended by the Ministry are useful)

Factor 2: Concerns about Vaccine Safety

- I5: "I nuovi vaccini comportano più rischi dei vecchi vaccini" (New vaccines carry more risks than old vaccines)
- I9: "Sono preoccupato per i gravi effetti avversi dei vaccini" (I am worried about serious adverse effects of vaccines)
- I10: "Non ho bisogno di vaccini per malattie che non sono più comuni" (I do not need vaccines for diseases that are no longer common)

Factor 3: Trust in Health Authorities and Compliance

- I6: "Le informazioni che ricevo sui vaccini dal CDC sono affidabili e degne di fiducia" (The information I receive about vaccines from the CDC is reliable and trustworthy)
- I7: "Accedere ai vaccini è un buon modo per proteggermi dalle malattie" (Accessing vaccines is a good way to protect myself from diseases)
- I8: "Generalmente, eseguo le indicazioni del mio medico curante a riguardo ai vaccini" (Generally, I follow my doctor's recommendations about vaccines)

These labels reflect the content of the items that load highly on each factor, capturing the underlying themes represented by each set of items.

Internal consistency

McDonald's Omega values were calculated for each of the three identified factors.

Factor 1 demonstrated a high reliability with an Omega value of approximately 0.92. This indicates that the items associated with Factor 1 are consistently measuring the underlying construct.

Factor 2 also showed good reliability with an Omega value of approximately 0.85, suggesting that the items related to this factor are reliable in their measurement.

Lastly, Factor 3 exhibited an Omega value of approximately 0.81, indicating acceptable reliability for the items within this factor.

Overall, the reliability estimates for all three factors suggest that the measurement model is sound and the items are dependable in capturing the constructs of interest.

SCORING PROCEDURE

For each respondent, calculate the mean response for the items loading onto each factor. This involves summing the responses to the relevant items and dividing by the number of items in that factor.

For Factor 1, compute the mean of VHS1, VHS2, VHS3, and VHS4.

For Factor 2, compute the mean of VHS5, VHS9, and VHS10.

For Factor 3, compute the mean of VHS6, VHS7, and VHS8.

Negatively worded items must be reverse-scored to ensure consistency in the scoring procedure. This process involves transforming the responses so that their directionality matches that of positively worded items. The negatively worded items in the Italian version of the include:

- Item 5: "I nuovi vaccini comportano più rischi dei vecchi vaccini" (New vaccines carry more risks than old vaccines)
- Item 9: "Sono preoccupato per i gravi effetti avversi dei vaccini" (I am worried about the serious adverse effects of vaccines)
- Item 10: "Non ho bisogno di vaccini per malattie che non sono più comuni" (I do not need vaccines for diseases that are no longer common)

These items should be reversed before scoring to ensure that higher scores consistently indicate more positive attitudes toward vaccines.
